# Psychological Symptoms in Primary Immunodeficiencies: a Common Comorbidity?

**DOI:** 10.1007/s10875-022-01207-7

**Published:** 2022-01-19

**Authors:** Olivia R. Manusama, Nico J. M. van Beveren, P. Martin van Hagen, Hemmo A. Drexhage, Virgil A. S. H. Dalm

**Affiliations:** 1 Department of Immunology, Erasmus University Medical Center, Rotterdam, The Netherlands; 2 Department of Psychiatry, Erasmus University Medical Center, Rotterdam, The Netherlands; 3 Department of Neuroscience, Erasmus University Medical Center, Rotterdam, The Netherlands; 4 Parnassia Group for Mental Health Care, Rotterdam/The Hague, The Netherlands; 5 Division of Allergy & Clinical Immunology, Department of Internal Medicine, Erasmus University Medical Center, Rotterdam, The Netherlands; 6 Academic Center for Rare Immunological Diseases (RIDC), Erasmus University Medical Center, Rotterdam, The Netherlands

To the Editor,

Primary immunodeficiency diseases (PIDs) encompass a heterogeneous group of rare disorders, characterized by an increased susceptibility to infections, autoimmune complications, autoinflammatory diseases, and malignancies [1]. Apart from these well-recognized disease manifestations, PID patients also suffer from psychological distress [2], significantly impacting their quality of life. Psychological well-being is currently under-researched in patients with PIDs. Moreover, the prevalence of psychological symptoms in these patients remains unclear. Therefore, we aimed to gain more insight into the disease burden and the nature of psychological complaints in a cohort of adult patients with PIDs.

We performed a cross-sectional study in 293 adult patients with PIDs, who were under treatment at the Primary Immunodeficiency Center of the Department of Internal Medicine, Erasmus University Medical Center Rotterdam, the Netherlands, for an established diagnosis of PID according to the International Union of Immunological Societies (IUIS) [1]. The study was conducted between April and June 2018 using the 4-Dimensional Symptom Questionnaire (4-DSQ), a self-reported questionnaire which measures four symptom dimensions (distress, depression, anxiety, and somatization) over 50 subitems. For each item, participants informed researchers whether they suffered from a specific symptom in the past week, and if yes, how often. This resulted in a score for each of the four dimensions, with different score ranges per dimension. The complete questionnaire has been included in the supplementary material (Suppl. doc. 1).

We compared the prevalence of psychological symptoms in patients with PID to the prevalence in the Dutch general population, using open-access data from the Longitudinal Internet Studies for the Social Sciences (LISS) panel, available online (https://www.dataarchive.lissdata.nl). We used one of the available samples (*n* = 4874) that was previously used to construct the normative 4DSQ scores, from which randomly two sex- and age-matched controls per patient were selected.

To evaluate whether patient-related factors are associated with these psychological symptoms, we retrieved clinical data from the electronic medical records, including immunological diagnosis, the presence of autoimmune manifestations, and laboratory results of immunoglobulin G (IgG) levels before the start of immunoglobulin replacement therapy. Moreover, self-reported data on life-time mental health treatment, psychiatric diagnosis, prior admission to a psychiatric ward, use of psychiatric and recreational drugs and alcoholic beverages, and family history of psychiatric disorders were collected.

Chi-squared tests were used for the individual 4DSQ items. The Mann–Whitney U test was used for aggregate scores. We performed four hierarchical multiple regression analyses to study associations between clinical factors and psychological symptoms.

The significance level was set at *P* < 0.05 for all statistical tests. SPSS Statistics version 24 and 25 (IBM, NY, USA) were used for statistical analyses.

Of 293 patients, 176 patients (60%) responded**.** Table S1 shows the characteristics of the respondents, hereinafter referred to as the study population. Primary antibody deficiency disorders were the most prevalent diagnoses in the study population (see Table S2). Almost half of the study population had received consultation or treatment for mental health issues (*n* = 80; 45.5%), of which 48 patients had been formally diagnosed with a psychiatric disorder. Table S3 summarizes the comparison of the 4DSQ scores of patients with PIDs with the Dutch control population. Cronbach’s alpha for internal consistency per dimension of the 4DSQ ranged between 0.89 and 0.95. For 47 out of 50 items, a significant difference in scores was observed between patients with PIDs and the general population. Moreover, patients specifically suffered from a “regular or constant” psychological symptom more often than controls, whereas the frequency of reporting “sometimes” was comparable between patients and controls. On all four dimensions, PID patients scored significantly higher, as shown in Table 1 and Figure S1. Evaluating the frequencies of “moderate” and “high” scores together — which are both considered to be aberrant — results in prevalence of symptom dimensions of patients with PID (33.9% distress, 18.9% depression, 22.4% anxiety, and 36.2% somatization) and controls (16.3% distress, 5.7% depression, 8.0% anxiety, and 11.2% somatization). The hierarchical regression models for the 4DSQ dimensions are summarized in Table S4. We found a significant association between two covariates (lifetime treatment for a mental health disorder and current use of psychiatric medication) and the presence of distress, depression, anxiety, and somatization. Overall, this study indicates the presence of a broad range of psychological symptoms in PID patients rather than specific symptoms or a certain symptom dimension. We did not find a higher prevalence of three of the 50 items scored: the occurrence of flashbacks, perception of indistinct threat, and the occurrence of repetitive behavior. The first two items are associated with anxiety and traumatic stress, whereas repetitive behavior is associated with obsessive–compulsive disorder. Our findings are in line with previous reports showing that mental health is compromised in patients with PIDs [2–4].

**Table 1:** 4DSQ dimension frequencies of PID patients and controls by conventional cutoffs

4DSQ scale	Score range	PID patients (*n* = 174)		Controls (*n* = 348)		*χ*^2^	*p*
		*n*	%	*n*	%		
Distress						24.6	< .001
* Low* *Moderate* *High*	0–10 11–20 21–32	115 36 23	66.1 20.7 13.2	291 44 13	83.6 12.6 3.7		
Depression						22.5	< .001
*Low* *Moderate* *High*	0–2 3–5 6–12	140 14 18	81.4 8.1 10.5	328 12 8	94.3 3.4 2.3		
Anxiety						25.9	< .001
*Low* *Moderate* *High*	0–3 4–9 10–24	135 23 16	77.6 13.2 9.2	320 23 5	92.0 6.6 1.4		
Somatization						54.6	< .001
*Low* *Moderate* *High*	0–10 11–20 21–32	111 43 20	63.8 24.7 11.5	309 36 3	88.8 10.3 0.9		

Higher scores indicate higher levels of symptoms. Both “moderate” and “high” scores are considered to be aberrant

There has been a growing interest in the relationship between psychiatric disorders and immune disturbances in psychiatric research [5]**.** These relationships have usually been investigated by starting with phenotypically defined psychiatric disorders and subsequently investigating the presence or absence of immune disturbances in patients compared to healthy controls. Here, we approached the immune-psychiatry relationship from the opposite direction by investigating the presence or absence of mental disturbances in patients with established immune deficits.

Patients with PIDs experience somatic health impediments, such as increased susceptibility to infections, autoimmunity, allergy, malignancy [1], and long-term treatment from early life, which most likely impact their mental well-being. There are thus several psychological mechanisms through which patients with PIDs are at risk for mental health difficulties, including fear of infections, social isolation, fatigue, maladaptation to illness, concerns over the future impact of illness, and unavailability of good coping strategies. These mechanisms fit within the psychosocial paradigm of mental health problems. On the other hand, the biological approach to mental health focuses on the direct relationship between biological abnormalities and disease processes in the brain. With regard to PID, in this framework, the biological aberrancies within the immune system may cause altered brain functioning, ultimately manifesting itself as mental dysfunction. This hypothesis is supported by the role of the immune system in brain development and plasticity and various immune implications in the pathogenesis of psychiatric disorders [5]. In any case, a complex, possibly bidirectional interplay between psychological, immunological, and neurological mechanisms exists. Since our study is cross-sectional, it was not designed to solve the cause and effect and dissect the psychological factors from biologically mediated processes. Further translational research is warranted to elucidate potential immunologic contributions to the development of psychopathology.

From a clinical point of view, PID patients suffer from substantial psychological symptoms, and patient-centered, personalized care should be modified in order to address this issue. We recommend active screening for psychological complaints after a diagnosis of PID is established and at regular time points during treatment. An important predictive factor, as supported by our results, is the previous mental health treatment, which should be consistently taken into account when taking the patient’s history. We also suggest involving a psychologist in the multidisciplinary care team for PID patients.

To the best of our knowledge, this study presents the first elaborate assessment of psychological complaints in a large cohort of patients with PID*.* We consider the use of age- and sex-matched controls as an additional improvement. However, this study has several limitations. First, a possible non-response bias might play a role, which could assert an effect in multiple directions. On the one hand, patients who suffer from psychological symptoms may be more likely to participate in the study as compared to patients who do not. On the other hand, patients who experience more psychological symptoms may be impeded from participation. Second, considering that most patients suffer from definite somatic morbidity, it is difficult to evaluate the presence of somatization symptoms precisely. However, we wish to emphasize that clinicians should be aware of the possibility that physical complaints arise from underlying psychological mechanisms in patients with PID. Finally, considering the exploratory nature of this study, no multiple test corrections were performed.

This study adds to the limited body of empirical evidence that a high level of psychological symptoms exists among adult PID patients. Regardless of whether these are emotional sequelae of chronic, multisystem illness or neuropsychological manifestations of immune dysregulation, psychological symptoms should be recognized and attended to, in order to improve PID patients’ health-related quality of life.

## Supplementary Information

Below is the link to the electronic supplementary material.

Supplementary file1 (DOCX 61 KB)

Supplementary file2 (PDF 602 KB)

Supplementary file3 (DOCX 340 KB)

## Data Availability

The datasets generated during and/or analyzed during the current study are available from the corresponding author on reasonable request.
