# Supplementary material for: Psychological Symptoms in Primary Immunodeficiencies: a Common Comorbidity?
Source: J Clin Immunol. 2022 Jan 19;42(3):695–8. doi: 10.1007/s10875-022-01207-7 (PMC9016014; doi:10.1007/s10875-022-01207-7)
Supplement: Supplementary file 1 — Supplementary file1 (DOCX 61 KB) [file 10875_2022_1207_MOESM1_ESM.docx]

***Supplemental Tables***

Article:

Psychological symptoms in primary immunodeficiencies: a common comorbidity?

Journal:

Journal of Clinical Immunology

Authors:

Olivia R. Manusama, Nico J. M. van Beveren, P. Martin van Hagen, Hemmo A. Drexhage, Virgil A. S. H. Dalm

Corresponding author:

Virgil A. S. H. Dalm

Department of Immunology, Erasmus University Medical Center Rotterdam, The Netherlands

Division of Allergy & Clinical Immunology, Department of Internal Medicine, Erasmus University Medical Center Rotterdam, The Netherlands

Academic Center for Rare Immunological Diseases (RIDC), Erasmus University Medical Center Rotterdam, The Netherlands

[v.dalm@erasmusmc.nl](mailto:v.dalm@erasmusmc.nl)

| Total no. respondents | 176 |
| --- | --- |
| Male : female | 68 : 108 (38.6% : 61.4%) |
| Age in years  Mean (SD) | 52.7 (15.9) |
| Immunodeficiency diagnosis |  |
| CVID | 76 (43.2%) |
| Isolated IgG subclass deficiency | 40 (22.7%) |
| SPAD | 15 (8.5%) |
| XLA | 3 (1.7%) |
| Isolated hypogammaglobulinemia | 10 (5.7%) |
| Other | 32 (18.2%) |
| IgG level (g/L) prior to treatment  Mean (SD) | 6.56 (3.84) |
| Autoimmune features | 46 (26.1%) |
| Psychiatric disorder | 48 (27.3%) |
| Neurodevelopmental disorders | 3 (1.7%) |
| Schizophrenia spectrum and other psychotic disorders | 3 (1.7%) |
| Bipolar and related disorders | 2 (1.1%) |
| Depressive disorders | 27 (15.3%) |
| Anxiety disorders | 9 (5.1%) |
| Obsessive-compulsive and related disorders | 2 (1.1%) |
| Trauma- and stressor-related disorders | 11 (6.3%) |
| Somatic symptom and related disorders | 1 (0.6%) |
| Gender dysphoria | 2 (1.1%) |
| Substance-related and addictive disorders | 1 (0.6%) |
| Personality disorders | 3 (1.7%) |
| Life-time mental health treatment | 80 (45.5%) |
| Prior admission at psychiatric ward | 9 (5.1%) |
| Current use of psychiatric medication | 39 (22.2%) |
| Antidepressants | 25 (14.2%) |
| Antipsychotics | 12 (6.8%) |
| Benzodiazepines | 18 (10.2%) |
| Stimulants | 2 (1.1%) |
| Anticonvulsants | 3 (1.7%) |
| Family history of psychiatric disorder | 39 (22.2%) |
| Average units of alcohol per week  Mean (SD) | 3.1 (5.7) |
| Current recreational drug use | 4 (2.3%) |

**Table S1. Characteristics of respondents.** CVID, common variable immunodeficiency; SPAD, selective polysacharide antibody deficiency; XLA, X-Linked Agammaglobulinemia

| **Immunological diagnosis** | **Number of patients** |
| --- | --- |
| Common variable immunodeficiency | 76 |
| IgG subclass deficiency | 40 |
| Selective antibody deficiency with normal immunoglobulins | 15 |
| Hypogammaglobulinemia | 10 |
| Comèl-Netherton Syndrome | 7 |
| Chronic mucocutaneous candidiasis | 6 |
| Hyper IgE syndrome | 4 |
| X-linked agammaglobulinemia | 4 |
| Hyper IgM syndrome | 3 |
| IgA deficiency | 3 |
| CD4+ lymphocytopenia | 2 |
| Chronic granulomatous disease | 1 |
| Complement component 2 deficiency | 1 |
| DiGeorge syndrome | 1 |
| Neutrophil chemotaxis defect | 1 |
| Polyglucosan body myopathy type 1 | 1 |
| X-linked moesin-associated immunodeficiency | 1 |

**Table S2. Immunological diagnoses of respondents.**

| **Symptom Scale** | **Patients (n = 176)** | | | **Controls (N = 348)** | | | ***P-value*** | ***χ²*** |
| --- | --- | --- | --- | --- | --- | --- | --- | --- |
| **Distress** | **No** | **Sometimes** | **Regularly, often or constantly** | **No** | **Sometimes** | **Regularly, often or constantly** |  |  |
| Q17 Feeling down or depressed | 111 (63.8%) | 35 (20.1%) | 28 (16.1%) | 253 (72.7%) | 77 (22.1%) | 18 (5.2%) | .000 | 17.234 |
| Q19 Suffering from worry | 71 (41.3%) | 56 (32.6%) | 45 (26.2%) | 179 (51.4%) | 121 (34.8%) | 48 (13.8%) | .002 | 12.484 |
| Q20 Suffering from disturbed sleep | 64 (36.8%) | 58 (33.3%) | 52 (29.9%) | 157 (45.1%) | 144 (41.4%) | 47 (13.5%) | .000 | 20.252 |
| Q22 Suffering from lack of energy | 103 (59.5%) | 36 (20.8%) | 34 (19.7%) | 257 (73.9%) | 71 (20.4%) | 20 (5.7%) | .000 | 24.995 |
| Q25 Feeling tense | 86 (49.4%) | 52 (29.9%) | 36 (20.7%) | 199 (57.2%) | 110 (31.6%) | 39 (11.2%) | .013 | 8.650 |
| Q26 Feeling easily irritated | 96 (55.2%) | 49 (28.2%) | 29 (16.7%) | 199 (57.2%) | 122 (35.1%) | 27 (7.8%) | .006 | 10.348 |
| Q29 Feeling unable to do anything anymore | 123 (71.9%) | 23 (13.5%) | 25 (14.6%) | 279 (80.2%) | 56 (16.1%) | 13 (3.7%) | .000 | 20.083 |
| Q31 Feeling unable to take interest in anyone/anything anymore | 133 (77.3%) | 21 (12.2%) | 18 (10.5%) | 296 (85.1%) | 47 (13.5%) | 5 (1.4%) | .000 | 22.195 |
| Q32 Feeling unable to cope anymore | 124 (72.1%) | 24 (14.0%) | 24 (14.0%) | 296 (85.1%) | 40 (11.5%) | 12 (3.4%) | .000 | 21.310 |
| Q36 Feeling unable to face it anymore | 131 (76.6%) | 23 (13.5%) | 17 (9.9%) | 312 (89.7%) | 30 (8.6%) | 6 (1.7%) | .000 | 22.376 |
| Q37 No longer feeling like doing anything | 122 (71.3%) | 27 (15.8%) | 22 (12.9%) | 289 (83.0%) | 47 (13.5%) | 12 (3.4%) | .000 | 17.924 |
| Q38 Having difficulty in thinking clearly | 117 (68.4%) | 29 (17.0%) | 25 (14.6%) | 284 (81.8%) | 53 (15.3%) | 10 (2.9%) | .000 | 26.230 |
| Q39 Having difficulty in getting to sleep | 93 (54.1%) | 42 (24.4%) | 37 (21.5%) | 207 (59.7%) | 99 (28.5%) | 41 (11.8%) | .014 | 8.530 |
| Q41 Easily becoming emotional | 95 (55.6%) | 45 (26.3%) | 31 (18.1%) | 224 (64.6%) | 96 (27.7%) | 27 (7.8%) | .002 | 12.537 |
| Q47 Having fleeting images of upsetting event(s) experienced in the past | 109 (63.4%) | 41 (23.8%) | 22 (12.8%) | 236 (68.0%) | 86 (24.8) | 25 (7.2%) | .112 | 4.377 |
| Q48 Having to do your best to put aside thoughts about upsetting event(s) | 117 (68.4%) | 35 (20.5%) | 19 (11.1%) | 281 (81.0%) | 47 (13.5%) | 19 (5.5%) | .005 | 10.779 |

| **Symptom Scale** | **Patients (n = 176)** | | | **Controls (N = 348)** | | | ***P-value*** | *χ²* |
| --- | --- | --- | --- | --- | --- | --- | --- | --- |
| **depression** | **No** | **Sometimes** | **Regularly, often or constantly** | **No** | **Sometimes** | **Regularly, often or constantly** |  |  |
| Q28 Feeling that everything is meaningless | 133 (78.2%) | 21 (12.4%) | 16 (9.4%) | 300 (86.2%) | 42 (12.1%) | 6 (1.7%) | .000 | 16.768 |
| Q30 Feeling that life is not worth while | 143 (83.6%) | 15 (8.8%) | 13 (7.6%) | 325 (93.4%) | 20 (5.7%) | 3 (0.9%) | .000 | 19.665 |
| Q33 Feeling that you would be better off if you were dead | 150 (87.2%) | 13 (7.6%) | 9 (5.2%) | 337 (96.8%) | 9 (2.6%) | 2 (0.6%) | .000 | 19.671 |
| Q34 Feeling that you cannot enjoy anything anymore | 134 (78.4%) | 19 (11.1%) | 18 (10.5%) | 313 (89.9%) | 30 (8.6%) | 5 (1.4%) | .000 | 23.915 |
| Q35 Feeling that there is no escape from your situation | 135 (78.9%) | 18 (10.5%) | 18 (10.5%) | 323 (92.8%) | 18 (5.2%) | 7 (2.0%) | .000 | 24.495 |
| Q46 Thinking “I wish I were dead” | 156 (90.7%) | 7 (4.1%) | 9 (5.2%) | 335 (96.5%) | 9 (2.6%) | 3 (0.9%) | .005 | 10.717 |

| **Symptom Scale** | **Patients (n = 176)** | | | **Controls (N = 348)** | | | ***P-value*** | *χ²* |
| --- | --- | --- | --- | --- | --- | --- | --- | --- |
| **anxiety** | **No** | **Sometimes** | **Regularly, often or constantly** | **No** | **Sometimes** | **Regularly, often or constantly** |  |  |
| Q18 Suffering from sudden fright for no reason | 138 (79.8%) | 23 (13.3%) | 12 (6.9%) | 313 (89.9%) | 30 (8.6%) | 5 (1.4%) | .001 | 14.575 |
| Q21 Suffering from a vague feeling of fear | 126 (72.8%) | 18 (10.4%) | 29 (16.8%) | 301 (86.5%) | 39 (11.2%) | 8 (2.3%) | .000 | 36.741 |
| Q23 Suffering from trembling when with other people | 154 (88.5%) | 12 (6.9%) | 8 (4.6%) | 336 (96.6%) | 8 (2.3%) | 4 (1.1%) | .001 | 13.200 |
| Q24 Suffering from anxiety or panic attacks | 143 (83.6%) | 19 (11.1%) | 9 (5.3%) | 337 (96.8%) | 9 (2.6%) | 2 (0.6%) | .000 | 29.501 |
| Q27 Feeling frightened | 125 (71.8%) | 28 (16.1%) | 21 (12.1%) | 306 (87.9%) | 33 (9.5%) | 9 (2.6%) | .000 | 26.124 |
| Q40 Having any fear of going out of the house alone | 150 (87.2%) | 12 (7.0%) | 10 (5.8%) | 337 (97.1%) | 6 (1.7%) | 4 (1.2%) | .000 | 19.597 |
| Q42 Being afraid of anything when there was really no need to be afraid | 147 (85.5%) | 10 (5.8%) | 15 (8.7%) | 320 (92.2%) | 23 (6.6%) | 4 (1.2%) | .000 | 18.695 |
| Q43 Being afraid to travel with public transport | 154 (90.1%) | 11 (6.4%) | 6 (3.5%) | 335 (96.5%) | 8 (2.3%) | 4 (1.2%) | .010 | 9.124 |
| Q44 Being afraid of becoming embarrassed when with other people | 145 (84.3%) | 16 (9.3%) | 11 (6.4%) | 306 (88.2%) | 34 (9.8%) | 7 (2.0%) | .037 | 6.584 |
| Q45 Feeling as if you were being threatened by unknown danger | 156 (90.7%) | 10 (5.8%) | 6 (3.5%) | 328 (94.5%) | 15 (4.3%) | 4 (1.2%) | .138 | 3.967 |
| Q49 Having to avoid certain places because they frightened you | 160 (93.6%) | 5 (2.9%) | 6 (3.5%) | 341 (98.3%) | 4 (1.2%) | 2 (0.6%) | .013 | 8.708 |
| Q50 Having to repeat some actions before you could do something else | 159 (93.0%) | 7 (4.1%) | 5 (2.9%) | 332 (95.7%) | 11 (3.2%) | 4 (1.2%) | .296 | 2.437 |

| **Symptom Scale** | **Patients (n = 176)** | | | **Controls (N = 348)** | | | ***P-value*** | *χ²* |
| --- | --- | --- | --- | --- | --- | --- | --- | --- |
| **somatization** | **No** | **Sometimes** | **Regularly, often or constantly** | **No** | **Sometimes** | **Regularly, often or constantly** |  |  |
| Q1 Suffering from dizziness or feeling light-headed | 108 (62.4%) | 38 (22.0%) | 27 (15.6%) | 267 (76.7%) | 66 (19.0%) | 15 (4.3%) | .000 | 22.095 |
| Q2 Suffering from painful muscles | 65 (37.8%) | 44 (25.6%) | 63 (36.6%) | 157 (45.1%) | 119 (34.2%) | 72 (20.7%) | .000 | 15.434 |
| Q3 Suffering from fainting | 162 (93.6%) | 7 (4.0%) | 4 (2.3%) | 342 (98.3%) | 1 (0.3%) | 5 (1.4%) | .003 | 11.402 |
| Q4 Suffering from neck pain | 94 (54.0%) | 32 (18.4%) | 48 (27.6%) | 223 (64.1%) | 83 (23.9%) | 42 (12.1%) | .000 | 19.702 |
| Q5 Suffering from back pain | 72 (42.1%) | 37 (21.6%) | 62 (36.3%) | 178 (51.1%) | 111 (31.9%) | 59 (17.0%) | .000 | 24.504 |
| Q6 Suffering from excessive sweating | 103 (59.5%) | 26 (15.0%) | 44 (25.4%) | 273 (78.4%) | 51 (14.7%) | 24 (6.9%) | .000 | 36.159 |
| Q7 Suffering from palpitations | 115 (66.5%) | 31 (17.9%) | 27 (15.6%) | 295 (84.8%) | 41 (11.8%) | 12 (3.4%) | .000 | 30.886 |
| Q8 Suffering from headache | 72 (41.6%) | 62 (35.8%) | 39 (22.5%) | 199 (57.2%) | 115 (33.0%) | 34 (9.8%) | .000 | 19.103 |
| Q9 Suffering from a bloated feeling in the abdomen | 86 (49.4%) | 39 (22.4%) | 49 (28.2%) | 250 (71.8%) | 77 (22.1%) | 21 (6.0%) | .000 | 51.408 |
| Q10 Suffering from blurred vision or spots in front of your eyes | 105 (61.0%) | 35 (20.3%) | 32 (18.6%) | 291 (83.6%) | 46 (13.2%) | 11 (3.2%) | .000 | 44.660 |
| Q11 Suffering from shortness of breath | 86 (49.7%) | 38 (22.0%) | 49 (28.3%) | 302 (86.6%) | 32 (9.2%) | 14 (4.0%) | .000 | 91.780 |
| Q12 Suffering from nausea or an upset stomach | 108 (62.4%) | 29 (16.8%) | 36 (20.8%) | 290 (83.3%) | 43 (12.4%) | 15 (4.3%) | .000 | 40.369 |
| Q13 Suffering from pain in the abdomen or stomach area | 107 (61.5%) | 41 (23.6%) | 26 (14.9%) | 282 (81.0%) | 54 (15.5%) | 12 (3.4%) | .000 | 31.122 |
| Q14 Suffering from tingling in the fingers | 125 (72.7%) | 24 (14.0%) | 23 (13.4%) | 277 (79.6%) | 52 (14.9%) | 19 (5.5%) | .008 | 9.713 |
| Q15 Suffering from pressure or a tight feeling in the chest | 128 (74.9%) | 29 (17.0%) | 14 (8.2%) | 313 (89.9%) | 33 (9.5%) | 2 (0.6%) | .000 | 29.990 |
| Q16 Suffering from pain in the chest | 145 (83.8%) | 20 (11.6%) | 8 (4.6%) | 330 (94.8%) | 14 (4.0%) | 4 (1.1%) | .000 | 17.656 |

**Table S3.** **4DSQ scores of PID patients vs. controls.** Q, question. Missing data accounts for the reduced N in a number of fields. The number of missing records of patients per item does not exceed 5.

Data from controls was obtained from <https://www.dataarchive.lissdata.nl>.

Reference: Scherpenzeel AC, Das M. “True” Longitudinal and Probability-Based Internet Panels: Evidence From the Netherlands. In: Das M, Ester P, Kaczmirek L, editors. Social and Behavioral Research and the Internet: Advances in Applied Methods and Research Strategies. Taylor and Francis Group, Boca Raton.; 2010. p. 77–104.

***Distress score***

|  | **b (95% CI)** | **SE_B_** | *ᵝ* | **p** |
| --- | --- | --- | --- | --- |
| **Step 1** |  |  |  |  |
| Constant | 11.15 (6.15, 16.14) | 2.53 |  | <.001 |
| Sex | -2.50 (-5.26, .28) | 1.40 | -.14 | .077 |
| Age | -.03 (-.11, .06) | .04 | -.05 | .533 |
| **Step 2** |  |  |  |  |
| Constant | 4.95 (.29, 9.61) | 2.36 |  | .037 |
| Sex | -1.12 (-3.54, 1.29) | 1.22 | -.06 | .360 |
| Age | .00 (-.07, .08) | .04 | .01 | .918 |
| Life-time mental health treatment | 6.62 (3.86, 9.38) | 1.40 | .37 | <.001 |
| Use of psychiatric medication | 4.58 (1.24, 7.92) | 1.69 | .21 | .007 |
| **Step 3** |  |  |  |  |
| Constant | 6.69 (1.59, 11.79) | 2.58 |  | .011 |
| Sex | -.99 (-3.40, 1.43) | 1.22 | -.05 | .421 |
| Age | -.00 (-.08, .08) | .04 | -.00 | .986 |
| Life-time mental health treatment | 6.64 (3.88, 9.39) | 1.39 | .37 | <.001 |
| Use of psychiatric medication | 4.45 (1.11, 7.78) | 1.69 | .21 | .009 |
| PID diagnosis | -.62 (1.36, .12) | .38 | -.11 | .101 |
| Autoimmunity | -.02 (-.96, .06) | .04 | -.03 | .630 |

*R^2^ = 0.19 for Step 1 (p = 0.193), ΔR^2^ = 0.26 for Step 2 (p < 0.001), ΔR^2^ = 0.01 for Step 3 (p = 0.225).*

***Depression score***

|  | **b (95% CI)** | **SE_B_** | *ᵝ* | **P** |
| --- | --- | --- | --- | --- |
| **Step 1** |  |  |  |  |
| Constant | 1.72 (-.09, 3.53) | .91 |  | .062 |
| Sex | -.27 (-1.28, .73) | .51 | -.04 | .594 |
| Age | -.00 (-.03, .03) | .02 | -.01 | .901 |
| **Step 2** |  |  |  |  |
| Constant | .16 (-1.63, 1.94) | .90 |  | .862 |
| Sex | .10 (-.83, 1.03) | .47 | .02 | .831 |
| Age | .01 (-.02, .03) | .01 | .02 | .744 |
| Life-time mental health treatment | 1.46 (.40, 2.52) | .54 | .23 | .007 |
| Use of psychiatric medication | 1.84 (.56, 3.12) | .65 | .24 | .005 |
| **Step 3** |  |  |  |  |
| Constant | .33 (-1.64, 2.29) | 1.00 |  | .745 |
| Sex | .12 (-.82, 1.06) | .47 | .02 | .799 |
| Age | .01 (-.03, .03) | .01 | .02 | .743 |
| Life-time mental health treatment | 1.46 (.39, 2.52) | .54 | .23 | .008 |
| Use of psychiatric medication | 1.81 (.52, 3.10) | .65 | .24 | .006 |
| PID diagnosis | -.06 (-.35, .23) | .15 | -.03 | .666 |
| Autoimmunity | 1.01 (-.04, .02) | .02 | -.04 | .590 |

*R^2^ = 0.002 for Step 1 (p = 0.865). ΔR^2^ = 0.166 for Step 2 (p < 0.001). ΔR^2^ = 0.003 for Step 3 (p = 0.780).*

***Anxiety score***

|  | **b (95% CI)** | **SE_B_** | *ᵝ* | **P** |
| --- | --- | --- | --- | --- |
| **Step 1** |  |  |  |  |
| Constant | 4.53 (1.86, 7.19) | 1.35 |  | .001 |
| Sex | -.22 (-1.70, 1.26) | .75 | -.02 | .769 |
| Age | -.04 (-.08, .01) | .02 | -.12 | .124 |
| **Step 2** |  |  |  |  |
| Constant | 2.00 (-.59, 4.60) | 1.31 | .04 | .129 |
| Sex | .40 (-.95, 1.74) | .68 | -.08 | .562 |
| Age | -.03 (-.07, .02) | .02 | .24 | .242 |
| Life-time mental health treatment | 2.22 (.69, 3.76) | .78 | .28 | .005 |
| Use of psychiatric medication | 3.21 (1.35, 5.07) | .94 | .04 | .001 |
| **Step 3** |  |  |  |  |
| Constant | 3.27 (.45, 6.09) | 1.43 | .05 | .024 |
| Sex | .49 (-.84, 1.83) | .68 | -.09 | .468 |
| Age | -.03 (-.07, .01) | .02 | .24 | .182 |
| Life-time mental health treatment | 2.24 (.71, 3.76) | .77 | .27 | .004 |
| Use of psychiatric medication | 3.12 (1.27, 4.96) | .93 | -.15 | .001 |
| PID diagnosis | -.45 (-.86, -.04) | .21 | -.04 | .032 |
| Autoimmunity | -.01 (-.05, .03) | .02 | .05 | .593 |

*R^2^ = 0.014 for Step 1 (p = 0.304). ΔR^2^ = 0.197 for Step 2 (p < 0.001). ΔR^2^ = 0.024 for Step 3 (p = 0.082).*

***Somatization Score***

|  | **b (95% CI)** | **SE_B_** | *ᵝ* | **P** |
| --- | --- | --- | --- | --- |
| **Step 1** |  |  |  |  |
| Constant | 12,14 (7.86, 16.41) | 2.16 |  | <.001 |
| Sex | -1,94 (-4.31, .43) | 1.20 | -.12 | .108 |
| Age | -0,04 (-.11, .04) | .04 | -.08 | .318 |
| **Step 2** |  |  |  |  |
| Constant | 8,59 (4.25, 12.93) | 2.20 |  | <.001 |
| Sex | -1,13 (-3.38, 1.12) | 1.14 | -.07 | .325 |
| Age | -0,02 (-.09, .05) | .04 | -.04 | .566 |
| Life-time mental health treatment | 3,54 (.97, 6.11) | 1.30 | .23 | .007 |
| Use of psychiatric medication | 3,30 (.19, 6.41) | 1.57 | .18 | .038 |
| **Step 3** |  |  |  |  |
| Constant | 11,54 (6.88, 16.21) | 2.36 | -.06 | <.001 |
| Sex | -0,96 (-3.17, 1.25) | 1.12 | -.07 | .392 |
| Age | -0,03 (-.10, .04) | .04 | .24 | .362 |
| Life-time mental health treatment | 3,60 (1.08, 6.11) | 1.27 | .17 | .005 |
| Use of psychiatric medication | 3,22 (.16, 6.27) | 1.54 | -.21 | .039 |
| PID diagnosis | -1,03 (-1.71, -.35) | .34 | .04 | .003 |
| Autoimmunity | 0,02 (-.05, .09) | .04 | -.06 | .575 |

*R^2^ = 0.019 for Step 1 (p = 0.20). ΔR^2^ = 0.127 for Step 2 (p < 0.001). ΔR^2^ = 0.045 for Step 3 (p = 0.011).*

B = unstandardized regression coefficient; SE_B =_ Standard error of the coefficient; ᵝ = standardized coefficient

**Table S4. Hierarchical Linear Regression with Coefficients (b), 95% Confidence Intervals (CI), Standard errors (SE), Standardized coefficients (ᵝ) and p-Values (*p*).** Confidence intervals and standard errors based on 1000 bootstrap samples. The outcome variables were distress, depression, anxiety, and somatization. In step 1, the predictive variables were sex and age. In Step 2, we introduced life-time mental health treatment and the use of psychiatric medication. In Step 3, we added type of PID diagnosis (common variable immunodeficiency (CVID), IgG subclass deficiency, selective polysaccharide antibody deficiency (SPAD), undefined hypogammaglobulinemia, or “other” and the presence of moderate to severe autoimmune complications.

The sociodemographic variables (sex and age) did not contribute significantly to explaining the variance in any of the regression analyses. The introduction of the “Life-time mental health treatment” and “Use of psychiatric medication” variable significantly increased the variance by 26% in the distress model, 16.6% in the depression model, 19.7% in the anxiety model, and 12.7% in the somatization model. Finally, “PID diagnosis” and “Autoimmunity” significantly increased the variance in somatization, but not in the other three models.
