# Supplementary material for: Psychological Symptoms in Primary Immunodeficiencies: a Common Comorbidity?
Source: J Clin Immunol. 2022 Jan 19;42(3):695–8. doi: 10.1007/s10875-022-01207-7 (PMC9016014; doi:10.1007/s10875-022-01207-7)
Supplement: Supplementary file 2 — Supplementary file2 (PDF 602 KB) [file 10875_2022_1207_MOESM2_ESM.pdf]

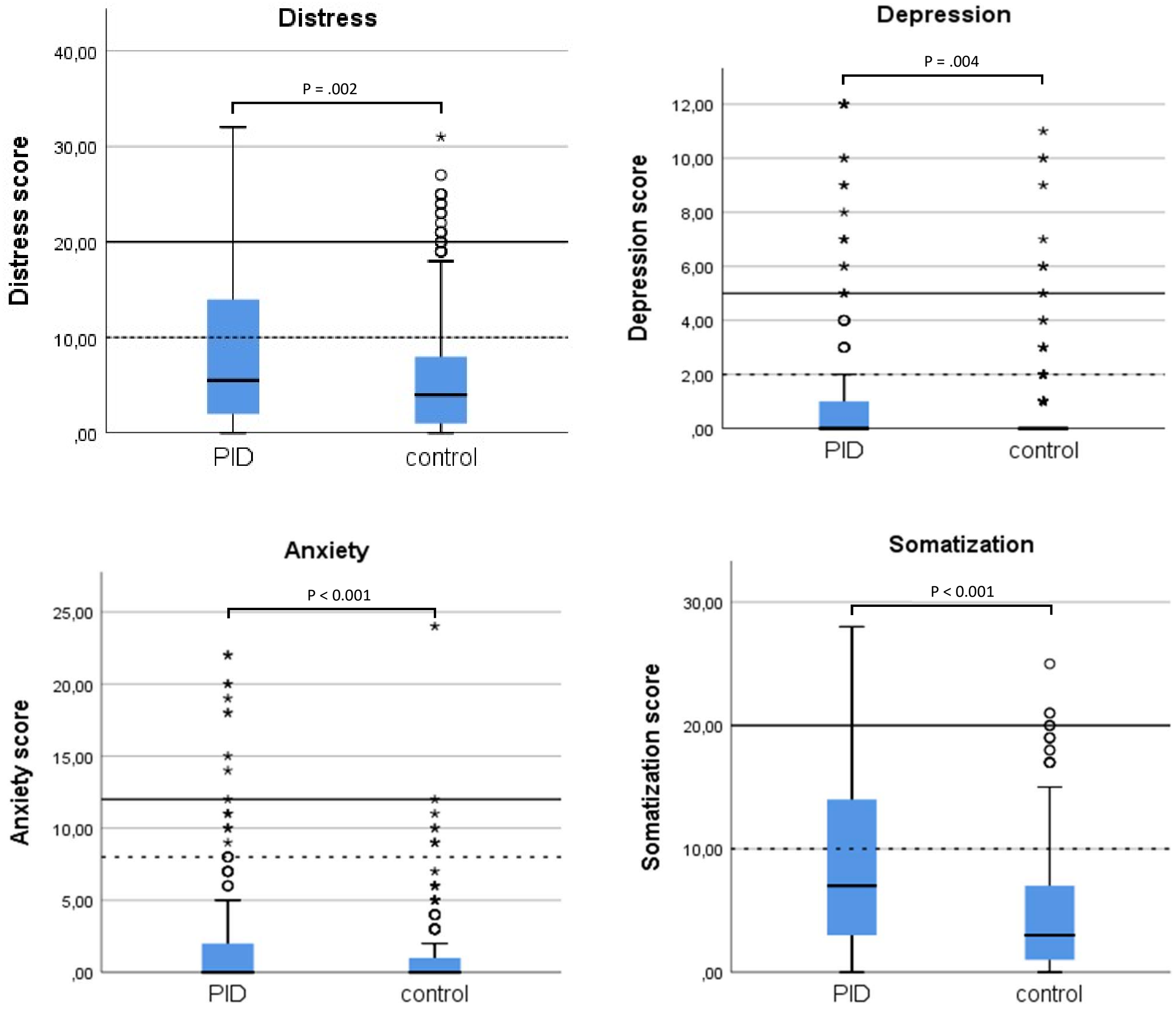

Fig. S1 Box plots showing Distress, Depression, Anxiety and Somatization scores in PID patients (N = 176) and controls (N = 348)

Score ranges: Distress (0-32), Depression (0-12), Anxiety (0-24), Somatization (0-32). Horizontal black lines within box represent median; upper and lower boundaries of box represent interquartile range; whiskers represent 75th and 25th percentile. Higher scores indicate higher levels of symptoms. Horizontal dash lines represent “moderately high” score and solid lines represent “very high” score; both are considered aberrant. Asterisks represent extreme scores and circles represent outliers. P value based on Mann-Whitney U test.

Data from controls was obtained from https://www.dataarchive.lissdata.nl.
Reference: Scherpenzeel AC, Das M. “True” Longitudinal and Probability-Based Internet Panels: Evidence From the Netherlands. In: Das M, Ester P, Kaczmirek L, editors. Social and Behavioral Research and the Internet: Advances in Applied Methods and Research Strategies. Taylor and Francis Group, Boca Raton.; 2010. p. 77–104.
