# Supplementary material for: Psychological Symptoms in Primary Immunodeficiencies: a Common Comorbidity?
Source: J Clin Immunol. 2022 Jan 19;42(3):695–8. doi: 10.1007/s10875-022-01207-7 (PMC9016014; doi:10.1007/s10875-022-01207-7)
Supplement: Supplementary file 3 — Supplementary file3 (DOCX 340 KB) [file 10875_2022_1207_MOESM3_ESM.docx]

**Suppl. Doc. 1: Four-Dimensional Symptom Questionnaire (4DSQ)**

Reference: Terluin B, Smits N, Brouwers EPM, de Vet HCW. The Four-Dimensional Symptom Questionnaire (4DSQ) in the general population: scale structure, reliability, measurement invariance and normative data: a cross-sectional survey. Health Qual Life Outcomes. 2016
